# Daily, weekly, and monthly dynamics of crowding in emergency department of university clinical centre Serbia: a one-year analysis of NEDOCS score

**DOI:** 10.1186/s12245-026-01313-y

**Published:** 2026-08-04

**Authors:** Slobodan Dzelebdzic, Bosiljka Djikanovic, Zoran Bukumiric, Natasa Maksimovic, Dusan Micic, Zorana Ana Rakočević, Vasa Curcin, Aleksandar Medarevic

**Affiliations:** 1 The Emergency Center, University Clinical Center of Serbia, Dr Subotica 8, Belgrade, 11000 Serbia; 2 Institute of Social Medicine, Faculty of Medicine, University of Belgrade, Belgrade, Serbia; 3 Centre - School of Public Health, Faculty of Medicine, University of Belgrade, Belgrade, Serbia; 4 Institute of Statistics and Medical Informatics, Faculty of Medicine, University of Belgrade, Belgrade, Serbia; 5 Department of Clinical Research, Faculty of Health Sciences, University of Southern Denmark, Denmark; 6 Institute of Epidemiology, Faculty of Medicine, University of Belgrade, Belgrade, Serbia; 7 Department of Population Health Sciences, King’s College London, London, SE1 1UL UK; 8 Institute of Public Health of Serbia “Dr Milan Jovanovic Batut”, Belgrade, Serbia

**Keywords:** Emergency department, Crowding, NEDOCS, Temporal patterns, Health services research, Health information systems, Operational management

## Abstract

**Background:**

Emergency department (ED) crowding is increasingly recognized as a global public health issue, while evidences from South-East Europe remain limited. The aim of this study was to characterize daily, annual, weekly, and monthly patterns of ED crowding in a high-volume tertiary hospital.

**Methods:**

We conducted a retrospective observational study of ED crowding in the largest ED in Serbia over 360 consecutive days (8,640 one-hour intervals). Crowding was measured hourly using the National Emergency Department Overcrowding Score (NEDOCS), derived from routinely collected data from health information system and expressed on a 0–200 scale, later grouped into six standard categories. Descriptive analyses assessed annual distribution, circadian and weekly variation, seasonal trends, and patient age structure. Between-group differences in NEDOCS scores were assessed using the Kruskal–Wallis test. Effect sizes were estimated using eta-squared (η²).

**Results:**

The majority of patients in ED (43.14%) were older than 60. Almost 90% of patients (89.5%) waited up to 4 h for the first check up by doctor. The median annual NEDOCS score was 43.87 (IQR 51.92 ), corresponding to a “busy” operational state. Level 2 (“busy”) occurred in 39.4% of hourly intervals and level 1 (“not busy”) in 29.5%, while overcrowding (levels 4–6) was observed in 5.6% of intervals. Crowding was significantly higher during daytime than nighttime hours (*p* < 0.001), with a pronounced morning peak. Weekday crowding exceeded weekend levels, peaking on Tuesdays. Seasonal variation showed higher crowding during winter and transitional months.

**Conclusions:**

ED crowding demonstrates predictable temporal patterns that mirror broader population health dynamics. Routine high-frequency monitoring may inform strategic staffing, adaptive capacity planning, and governance mechanisms aimed at strengthening emergency care performance and system resilience.

**Supplementary Information:**

The online version contains supplementary material available at 10.1186/s12245-026-01313-y.

## Introduction

Emergency department (ED) crowding is increasingly recognized as a public health and health system performance issue rather than solely an operational concern [1, 2]. It reflects imbalances between population health needs and available health care capacity, with potential implications for access, quality, patient safety, and equity [3]. Although crowding is often defined as a mismatch between demand for emergency care and available resources, its most salient characteristic is temporal variability. Patient arrivals, internal care processes, and inpatient bed availability fluctuate across hours, days, and seasons, creating recurrent patterns of operational strain shaped by epidemiological trends, organizational factors, and demographic change [4, 5].

Population ageing represents one of the most significant structural drivers of ED utilization in the world [6, 7]. Serbia is undergoing pronounced demographic transition, with a steady increase in the proportion of adults aged 65 years and older (currently, 22%) and a rising median population age [8]. This shift has important implications for emergency care demand. Older adults disproportionately use ED services due to multimorbidity, polypharmacy, functional decline, and acute exacerbations of chronic diseases [7]. These factors are associated with more complex presentations, longer diagnostic evaluation, prolonged length of stay, and higher hospital admission rates [9, 10]. Consequently, even stable arrival volumes may generate increased operational pressure because of greater clinical complexity and resource intensity.

Demographic change interacts with temporal demand patterns. Daily peaks may be influenced by referral pathways, emergency medical services activity, and care coordination across sectors. Weekly variability may reflect accessibility of primary and specialist care, while seasonal increases—particularly during winter—are frequently associated with respiratory infections and cardiovascular decompensations, especially among older adults [11]. These interacting dynamics underscore the need to understand ED crowding as a system-level phenomenon embedded within broader population health trends.

Despite extensive research on structural determinants of ED crowding, relatively few studies provide high-resolution longitudinal analyses capable of capturing circadian, weekly, and seasonal variability over extended periods [5, 12, 13]. Aggregated daily or monthly measures may obscure short-term fluctuations that are critical for staffing strategies, surge preparedness, and adaptive capacity planning.

Standardized instruments such as the National Emergency Department Overcrowding Score (NEDOCS) enable objective quantification of operational strain [14, 15]. Continuous hourly monitoring provides granular insight into predictable patterns of system pressure and supports data-informed governance of emergency care services.

Evidence from South-East Europe remains limited. In health systems experiencing demographic ageing and evolving patterns of service utilization, understanding the interaction between temporal variability and structural population change is essential for strengthening emergency care resilience and ensuring sustainable access.

The aim of this study was to characterize annual, weekly, and circadian dynamics of ED crowding in a high-volume tertiary emergency department, using hourly NEDOCS measurements over a one-year period, within the broader context of demographic transition and increasing utilization by older adults. By applying high-frequency routine health information system data, the study sought to identify predictable temporal patterns that may support staffing, operational planning, and emergency care management.

## Method

### Study design and setting

This was a retrospective observational study conducted at the ED of Emergency Center of the University Clinical Center of Serbia in Belgrade, Serbia. The center is a high-volume tertiary emergency department located in the capital city and serves as a major referral facility for adult emergency care. The analysis was based on aggregated hourly operational data extracted from the Heliant health information system (HIS), in an almost one-year period, from April 11, 2022, to April 5, 2023, since the last five days of that period in April (April 6–10, 2023), HIS was not fully functional and could not provide all needed data.

The study was conducted in accordance with the Declaration of Helsinki and approved by the Ethical Committee of the Faculty of Medicine University of Belgrade, Serbia, decision no. 17/IX-4. The study was based on aggregated, fully anonymized operational data. No individual patient data were accessed, and informed consent was not required.

### Assessment of crowding by NEDOCS score

As a measure of crowding we used and calculated NEDOCS score. Higher NEDOCS values indicate greater operational strain and a higher degree of ED crowding. NEDOCS variables were taken from routinely collected data available from the hospital health information system. Because the database did not provide a continuous real-time census of all patients physically present in the ED at each exact hour, the hourly number of ED patients was derived from patients registered in the system during each one-hour interval.

NEDOCS score takes into account seven variables, such as total number of beds in the ED (reception and observation capacity) (A), which, in this case, equals 50; total number of beds in the hospital (admission capacity), which, in this case, equals 388 (B); the total number of patients in the ED who opened medical history within an hour (C); the number of patients treated with mechanical ventilation i.e. the number of patients admitted to the Intensive Care Unit (ICU) within an hour (D); the length of stay (in hours) of patients who waited the admission to hospital for the longest time in ED (E); the number of the patients admitted to ward (F), and the longest patient waiting time from desk registration to check-up in that hour interval, expressed in hours (G) [14, 15]. NEDOCS score was calculated based on the formula: 85.8 (C/A) + 600 (F/B) + 13.4 (D) + 0.93 (E) + 5.64 (G) – 20 [14, 15]. Calculated score laid on the 0-200 scale. When NEDOCS score had a negative value (if in a given hour no patients appear, typically during the night, parameter G), we assigned it the value 0 (or category 1) to that hour (not busy). Calculated NEDOCS score was grouped into six categories: (1) 0–20 (not busy); (2) 21–60 (busy); (3) 61–100 (extremely busy but not overcrowded); (4) 101–140 (overcrowded); (5) 141–180 (severely overcrowded), and (6) 181–200 (dangerously overcrowded) [14, 15].

### Assessment of the most common diagnoses

For every one-hour interval, the three most common diagnoses assigned by the physician were reported from the health information system. Diagnoses were coded according to the International Classification of Diseases, 10th Revision (ICD-10) [16]. Diagnoses that appeared in at least 1% were reported in this study.

### Statistical analysis

Categorical variables were presented as frequencies and percentages. Continuous variables were summarized using median and interquartile ranges (IQRs). The distribution of hourly NEDOCS scores was assessed descriptively across predefined temporal categories, including hour of day, daytime versus nighttime shifts, day of the week, weekday versus weekend, and month.

The distribution of NEDOCS scores was examined across predefined temporal categories: hour of the day, day of the week, and month of the year. As NEDOCS scores were not assumed to follow a normal distribution, between-group differences were assessed using the Kruskal–Wallis test. Effect sizes were estimated using eta-squared (η²). When statistically significant differences were detected, post-hoc pairwise comparisons were conducted using Dunn’s test with a Bonferroni correction for multiple comparisons. A Bonferroni-adjusted p-value < 0.05 was considered statistically significant.

## Results

### General characteristics of the use of ED during one year: the number of visits, age structure and the most common diagnoses

**Fig. 1:**
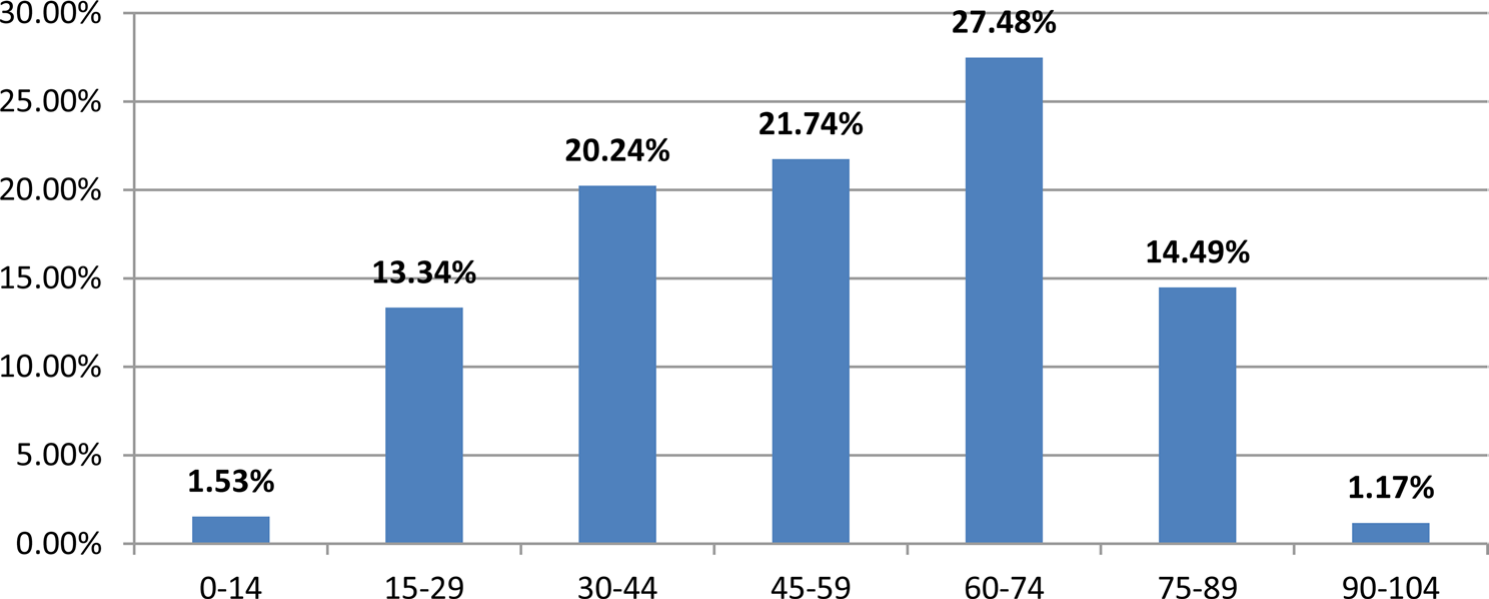
Distribution of patients by categorical age group in the ED of a tertiary care hospital between April 2022 and April 2023

During the 360-day observation period (8,640 one-hour intervals), when the health information system was fully operational, there were 178,679 visits. Patients 60–74 years old were the biggest group (27.5%), and the whole age structure is presented in Fig. 1.

The proportion of patients who waited up four hours was 89.5%, while more than one fifth, or 22%, waited up to one hour (Table 1).

**Table 1:** Distribution of the “longest waiting time” from desk registration time to doctor’s check-up, during one year (360 days)

	Frequency	%	Cumulative %
00–59 min	1903	22	22.0
60–119 min	3012	34.9	56.9
120–179 min	1994	23.1	80.0
180–239 min	827	9.6	89.5
240–299 min	370	4.3	93.8
300–359 min	195	2.3	96.1
360 min and longer	339	3.9	100.0
Total	8640	100.0	

Most common diagnoses were “other and unmarked stomach ache“ (18.4%) and “elevated blood pressure of unknown origin” (12.4%), along with a radiological reviews, unclassified”(31.9%), as a follow of the diagnostic procedure (Appendix 1). Diagnoses which were noted in a less than 1% of cases were not presented.

### Annual emergency department crowding measured by NEDOCS

**Fig. 2:**
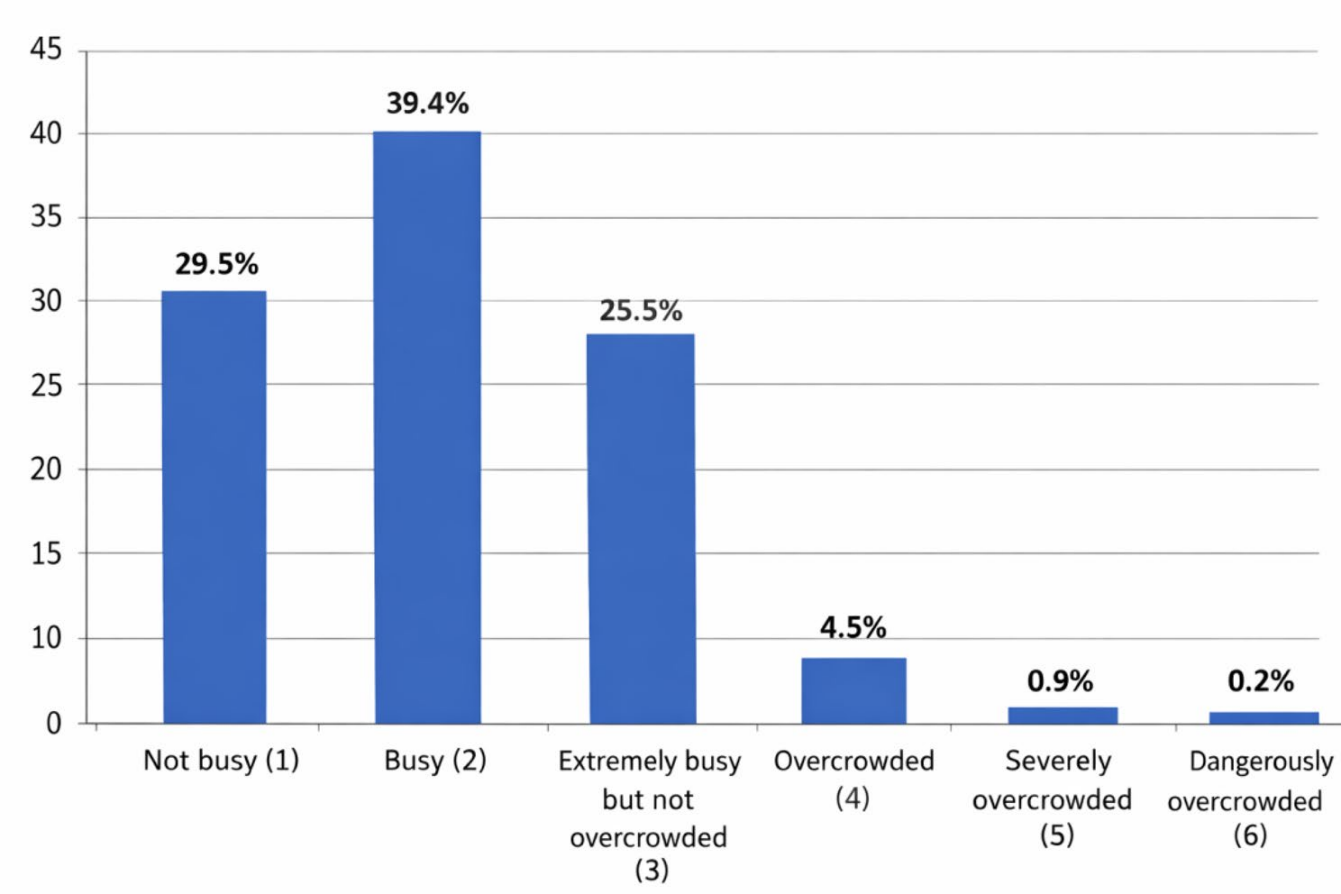
Distribution of hourly NEDOCS categories during the 360-days observation period

The median annual NEDOCS score was 43.87 (IQR 51.92), corresponding to the “busy” category and indicating a sustained operational load during the study period. Most hourly intervals fell within the lower and intermediate NEDOCS categories, particularly “busy,” “not busy,” and “extremely busy but not overcrowded” (Fig. 2). Overcrowded conditions, defined as NEDOCS categories 4–6, were observed in 5.6% of hourly intervals, while severely or dangerously overcrowded conditions were uncommon, occurring in 1.1% of intervals.

### Circadian variation (24-hour pattern)

**Fig. 3:**
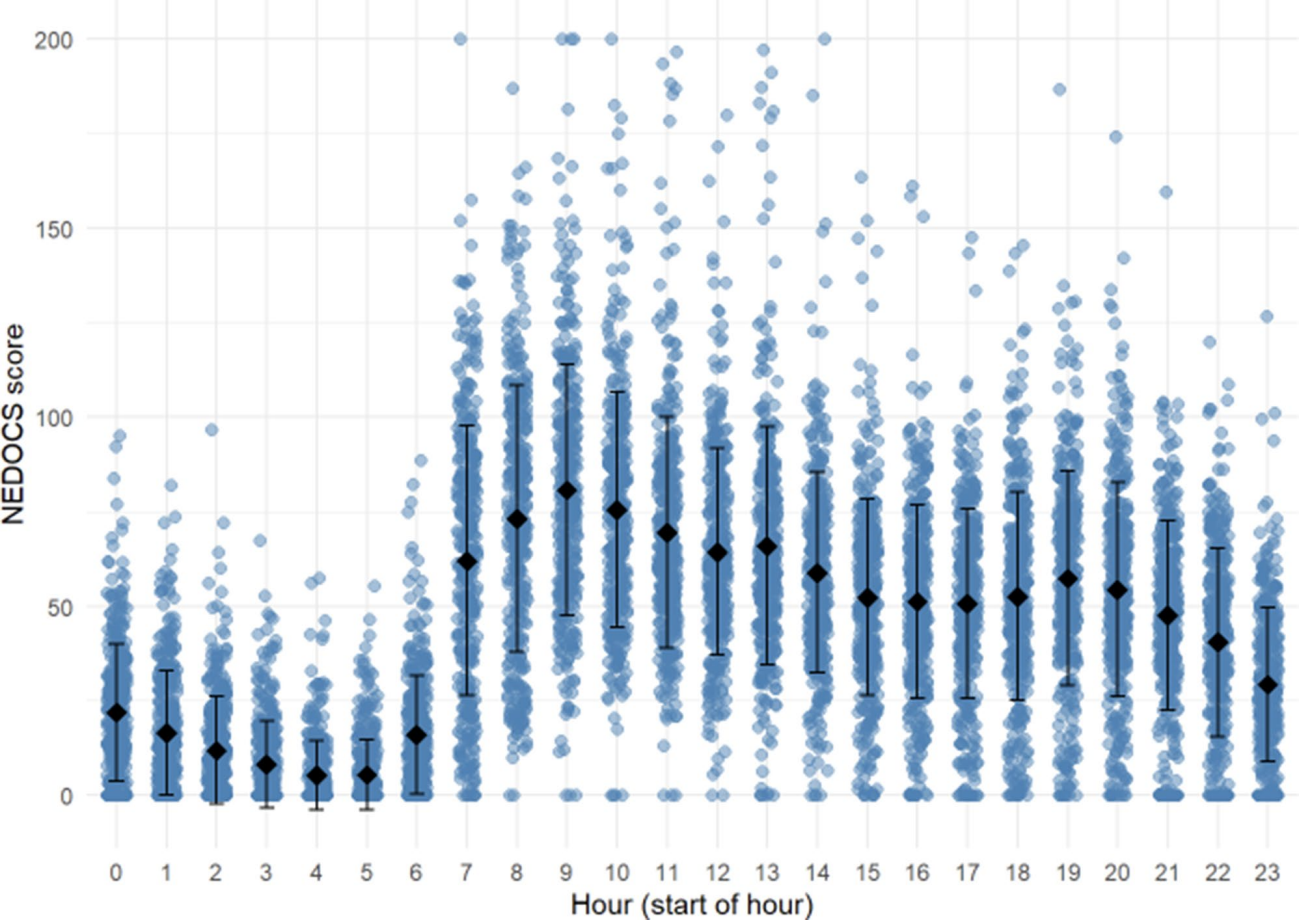
Distribution of the NEDOCS score within 24 h, throughout the year (plotted points represent means, joined with SDs, while drawn line on NEDOCS score at 100 indicates a threshold for crowding)

Hourly NEDOCS scores demonstrated a clear circadian pattern (Fig. 3). The highest values were observed during daytime and evening hours, particularly between 07:00 and 22:00, with the most prominent peak between 08:00 and 10:00 and the highest modal value in the 09:00–10:00 interval (median 78.3, IQR 42.7). The lowest NEDOCS values occurred during the night, particularly between 02:00 and 05:00, with the minimum observed in the 03:00–04:00 interval (median 0.0, IQR 7.4). A marked increase in NEDOCS scores was observed after 06:00.

NEDOCS scores differed significantly across hours of the day (Kruskal–Wallis χ²(23) = 4547.25, *p* < 0.001, η² = 0.5192). Post-hoc pairwise comparisons showed that 209 of 276 possible pairwise comparisons reached statistical significance after Bonferroni correction.

### Weekly variation

**Fig. 4:**
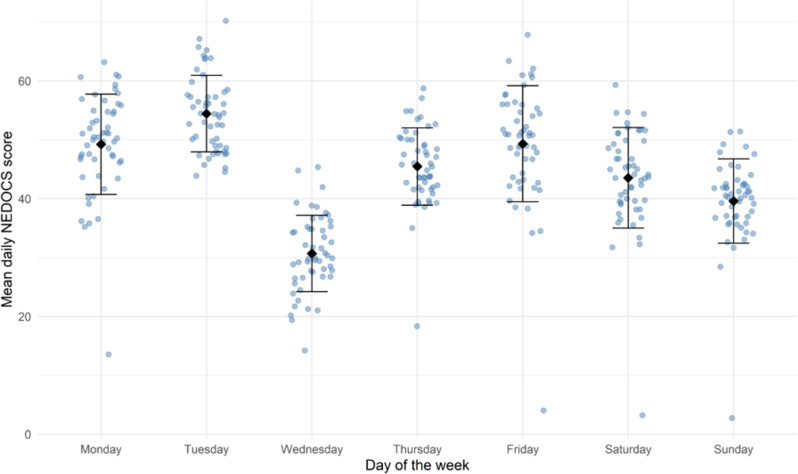
Average daily NEDOCS score by day of the week

Weekly analysis showed that the highest mean daily NEDOCS score was observed on Tuesday (median 54.3, IQR 8.8), followed by Friday, Monday, Thursday, Saturday, and Sunday, while Wednesday had the lowest mean values (median 29.9, IQR 8.2) (Fig. 4).

Mean daily NEDOCS scores differed significantly across days of the week (Kruskal–Wallis χ²(6) = 186.83, *p* < 0.001, η² = 0.5133). Dunn’s post-hoc pairwise comparisons with Bonferroni correction showed that 14 of 21 possible pairwise comparisons were statistically significant.

### Monthly variation

**Fig. 5:**
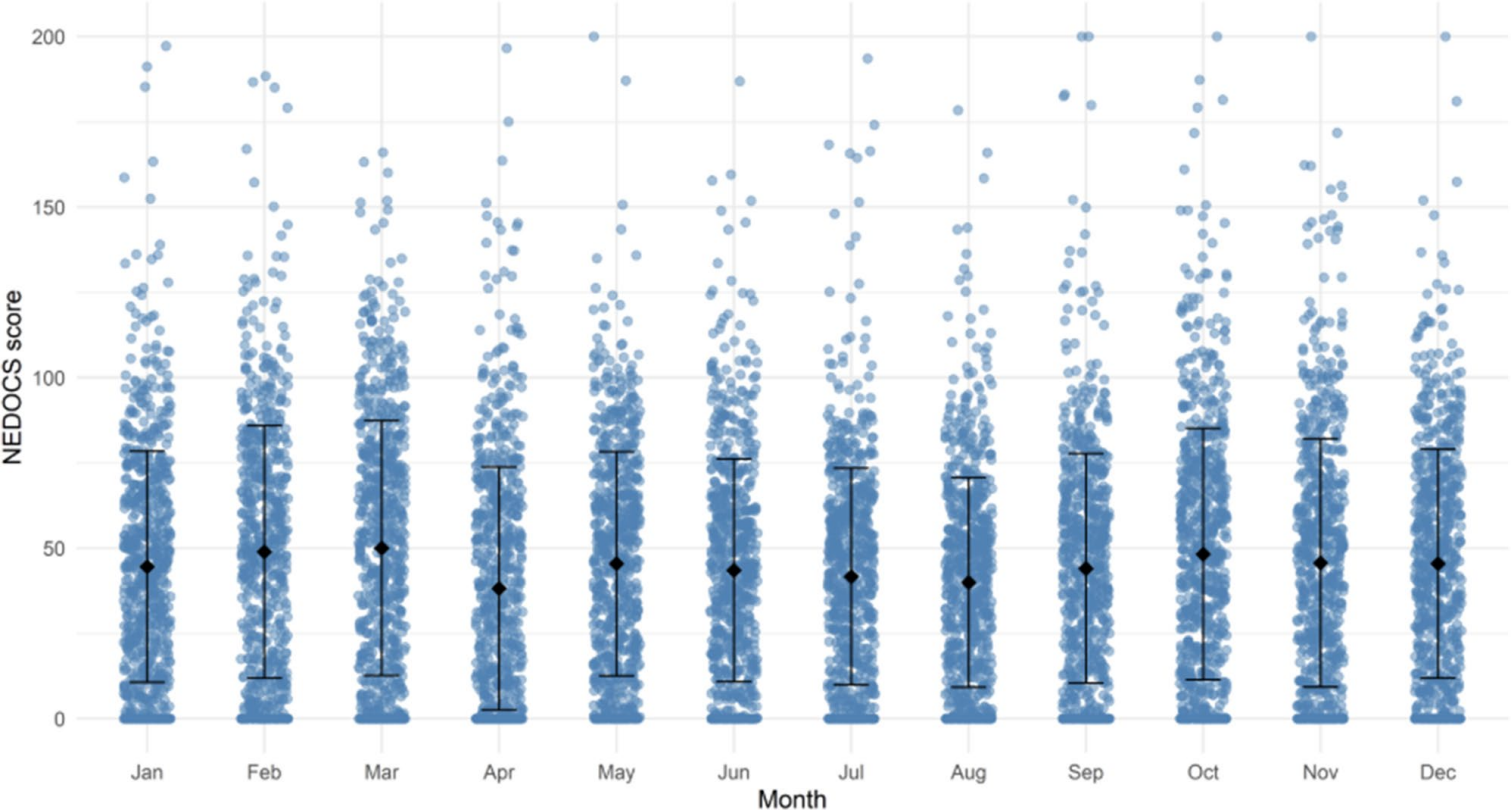
Distribution of the NEDOCS score by month of the year

**Fig. 6:**
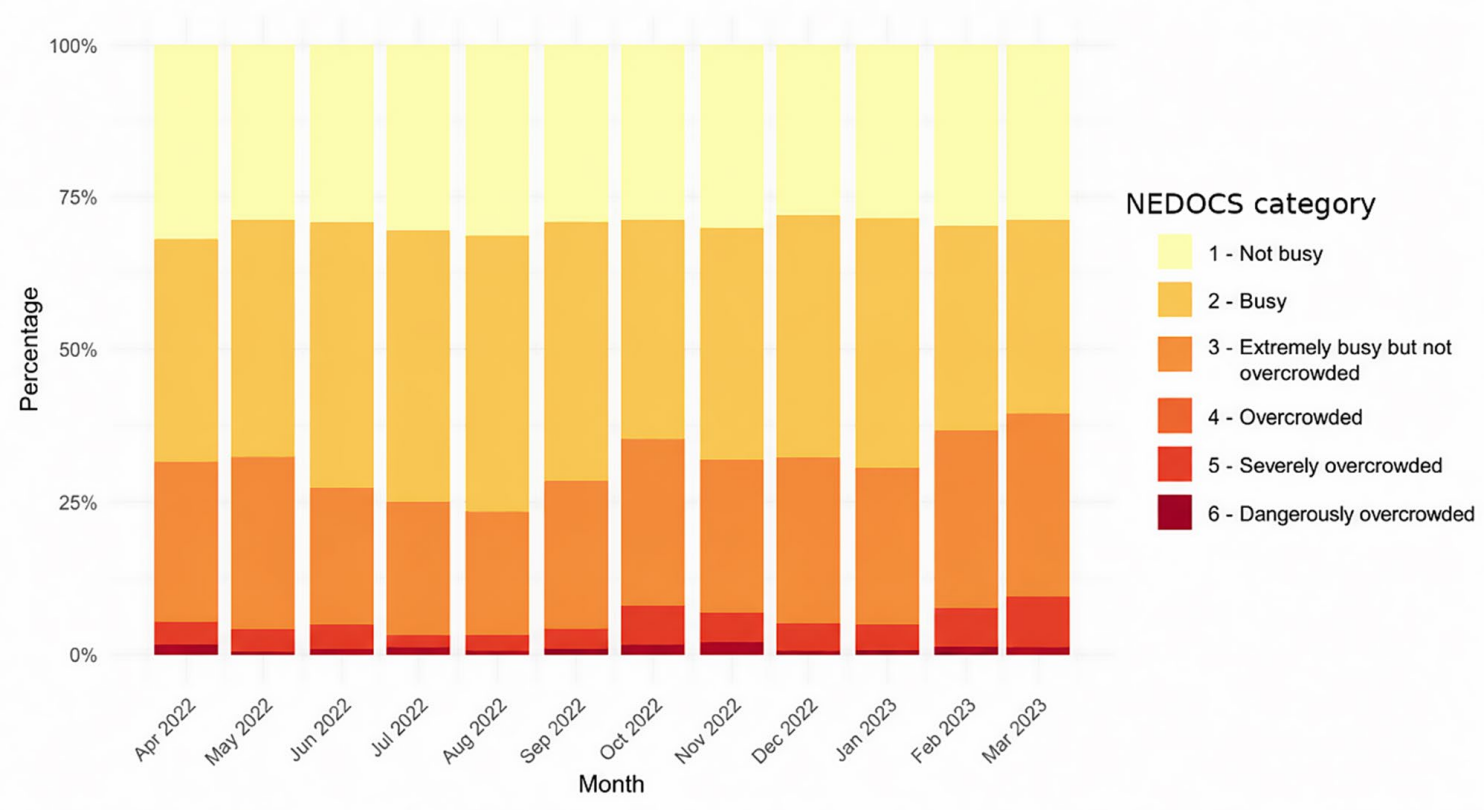
Monthly distribution of NEDOCS categories during the study period

Monthly analysis revealed clear seasonal variation. The highest mean NEDOCS values were recorded in autumn and winter time, from October to March (excluding January, which is a month with key religious holidays and days off, according to the Orthodox Julian Calendar) (Figs. 5 and 6). The peak was in March 2023 (mean 50, SD 37, IQR 60.9).

The lowest values were observed during summer months (June, July, and August), with August demonstrating the lowest overall crowding levels (mean 39.9, SD 30.8, IQR 44.9) The single lowest value was recorded in April, although data for this month were collected in two years (April 11–30, 2022, when ED started to work at the location, which might affected the number of patients, and the period of five days in April next year, April 1–5, 2023).

NEDOCS scores differed significantly across months of the year (Kruskal-Wallis χ²(11) = 84.27, *p* < 0.001, η² = 0.0096). Post-hoc pairwise comparisons were conducted using the Dunn test with Bonferroni correction, and out of 66 possible pairwise comparisons, 17 reached statistical significance (Bonferroni-adjusted *p* < 0.05).

## Discussion

This study provides a one-year, high-resolution description of ED crowding in the largest tertiary emergency center in Serbia using hourly NEDOCS measurements. The main findings were that the ED operated predominantly in the ‘busy’ category, that overcrowded conditions occurred in a minority of hourly intervals, and that crowding followed clear circadian, weekly, and monthly patterns. The highest crowding levels were observed during daytime hours, particularly in the morning, with additional variation across weekdays and months.

This study aimed to identify the levels of emergency department crowding in the largest tertiary emergency centre in Serbia during a whole year, for the first time, and we identified persistent and temporally structured patterns of the use of ED. Although extreme overcrowding was rare, the department functioned predominantly under moderate operational load, with clearly defined circadian, weekly, and seasonal peaks.

The age distribution of emergency department (ED) patients in our study indicates a pronounced predominance of older adults: older than 60 represent the proportion of 43.14% of all patients. This pattern aligns with broader demographic trends observed across Europe, including Serbia, where population ageing has accelerated over the past decades [8, 17]. Older adults tend to use emergency services more frequently due to the higher prevalence of multimorbidity, polypharmacy, frailty, and acute exacerbations of chronic diseases that require urgent clinical evaluation [18]. Previous studies have consistently shown that older ED patients present with more complex clinical conditions, require more diagnostic procedures, and experience longer lengths of stay compared with younger patients [19, 20]. Consequently, the substantial representation of patients aged 60 years and older observed in our sample may contribute to increased resource utilization and operational pressure in the ED [21]. Even when the overall number of visits remains stable, an older case-mix can prolong patient flow times and increase bed occupancy, thereby contributing to higher levels of crowding and elevated values of indicators such as the NEDOCS score.

While our data do not allow direct assessment of the contribution of older patients to ED crowding, the observed age structure indicates that population aging remains an important contextual factor for emergency care planning. Future patient-level analyses are needed to determine whether geriatric-sensitive triage, multidisciplinary assessment, and stronger integration between emergency, inpatient, and community-based care could help reduce crowding. Strong primary care capacity should serve as a prevention mechanism that keeps ED at tertiary level less occupied [1]. Recent analysis of the use of ED at the territory of the city of Belgrade identified that the greatest proportion of patients are coming to ED at University Clinical Center Serbia in their own (81.5%), which means they are bypassing prehospital (primary healthcare system) assessment of their health status [22]. This might indicate lower availability of these services and/or patients reluctance to use them timely, prior to exaggerating symptoms, which is a trend observed worldwide [23–25]. In transitional health systems in Central and Eastern Europe, EDs may function as compensatory entry points when primary and specialist care accessibility is variable [26]. With this in mind, practical recommendation would be to strengthen lower levels of health care services for better management of multimorbidities and chronic diseases, in order to prevent overcrowding and urgent care at the tertiary level [1].

The mean annual NEDOCS score corresponded to the “busy” category, indicating sustained pressure throughout the year. Severe overcrowding occurred in 5.6% of hourly intervals, which is similar to the crowding structure observed in Turkey [15], but more than in The Netherlands, where only 2.7% of the NEDOCS scores were in this category or higher, although assessed just over four weeks, not the whole year as we did [27].

From a public health perspective, ED crowding reflects broader system-level imbalances between population needs and available care pathways [1, 2, 12]. According to the widely cited input–throughput–output model proposed by Asplin et al. [3], crowding is shaped not only by patient arrivals, but also by internal care processes and inpatient bed availability.

The pronounced morning peak (08:00–10:00) and significantly higher daytime crowding (*p* < 0.001) indicate predictable circadian accumulation, and similar diurnal patterns have been reported in other high-volume emergency departments [28]. Weekday crowding exceeded weekend levels, with the highest mean values observed on Tuesdays. The regularity of the morning surge observed in this study suggests that anticipatory workforce alignment and improved cross-sector coordination may mitigate peak accumulation. However, that solution goes beyond reorganization of current capacities and requires more fundamental investment in the workforce, in the whole healthcare system.

Emergency departments worldwide are experiencing persistent understaffing, especially among nurses and emergency physicians, which is closely linked to poorer patient outcomes and declining staff well-being. A recent systematic review found that lower nurse staffing in emergency departments is associated with longer waiting times, increased length of stay, higher rates of patients leaving without being seen, delayed medications and interventions, and increased risk of in-department cardiac arrest [29]. Qualitative and survey studies show that emergency nurses frequently report inadequate staffing, unmanageable workload, and overtime as routine, leading to stress, burnout, and high intentions to leave [30]. Staff shortages are also a key contributor to emergency department overcrowding and high perceived workload, alongside rising patient inflow and barriers to patient throughput [30–32]. Literature review depicts understaffing as both a driver and amplifier of crowding, delays, and safety risks, underscoring the need for evidence-based staffing models and workforce retention strategies in emergency care [29–33].

Seasonal increases during winter and transitional months are consistent with established epidemiological patterns, particularly respiratory infections and cardiovascular exacerbations [34, 35].

Our findings showed that 89,5% of patients were evaluated within the first four hours upon checking in and opening medical history. Many health systems around the world (United Kingdom, Australia, New Zealand, Western Australia) use the 4-hour standard as a core benchmark for ED performance, which is linked to reduce overcrowding, faster assessment/admission, and sometimes better outcomes [36–39].

Integrating operational metrics with outcome indicators in future studies would provide a more comprehensive evaluation of crowding consequences. Embedding crowding surveillance within routine health system management may therefore contribute to improved adaptive capacity, particularly in ageing European health systems.

### Strengths and limitations

The main strength of this study is the use of a high-resolution dataset covering 8,640 consecutive one-hour intervals over 360 days, which allowed detailed assessment of temporal crowding patterns. The use of routinely collected health information system data also supports feasibility and reproducibility. However, in the calculation of the NEDOCS score, we used the number of patients who registered within an hour, rather than a real-time census of all patients physically present in the ED at that moment, which may have led to underestimation or misclassification of crowding intensity, especially during periods when many patients remained in the department across multiple hourly intervals.

Routine operational data may be affected by coding inconsistencies, missingness, and variability in documentation practices, particularly when it comes to diagnoses. In addition, the single-center design limits generalizability, and the use of aggregated hourly data prevented patient-level analyses of case mix, age-specific utilization, outcomes, and causal mechanisms. Future research should integrate crowding metrics with patient-based outcome data, and also should be scaled up to the other ED centres, which would allow comparisons and would strengthen evidences relevant for public health planning of ED resources.

## Conclusions

Hourly NEDOCS monitoring over a one-year period demonstrated clear circadian, weekly, and monthly patterns of ED crowding in a high-volume tertiary emergency department. Although dangerously overcrowded states were uncommon, the department operated under sustained operational pressure for much of the study period. Routine high-frequency monitoring of crowding may support evidence-informed staffing, operational planning, and adaptive capacity management in emergency care.

## Supplementary Information

Below is the link to the electronic supplementary material.

Supplementary Material 1

## Data Availability

Data sets used as a foundation for this manuscript might be available upon reasonable request.

## Abbreviations

ED: Emergency Department
HIS: Health Information System
NEDOCS: National Emergency Department Overcrowding Score
SD: Standard deviation
