# Supplementary material for: Daily, weekly, and monthly dynamics of crowding in emergency department of university clinical centre Serbia: a one-year analysis of NEDOCS score
Source: Int J Emerg Med. 2026 Aug 4;19:195. doi: 10.1186/s12245-026-01313-y (PMC13435447; doi:10.1186/s12245-026-01313-y)
Supplement: Supplementary file 1 [file 12245_2026_1313_MOESM1_ESM.docx]

Appendix 1. Most common diagnoses which were recorded at patients who have contacted ED

| **Diagnosis (code)** | **Interpretation** | **Percentage %** |
| --- | --- | --- |
| Z01.6 | Radiological review, unclassified | 31.9% |
| R10.4 | Other and unmarked stomach ache | 18.4% |
| I10 | Elevated blood pressure of unknown origin | 12.4% |
| W19.9 | Unmarked fall, unmarked places | 7.2% |
| Z04.9 | Overview and observation because of unmarked reason | 7.1% |
| Z98.8 | Other postoperative conditions | 3.0% |
| R07.4 | Chest pain | 1.4% |
| R51 | Headache | 1.3% |
| R42 | Instability and dizziness | 1.1% |
| S00.0 | Surface head injury | 1.0% |
